# Overexpression of microRNA-202-3p in bone marrow mesenchymal stem cells improves cerebral ischemia-reperfusion injury by promoting angiogenesis and inhibiting inflammation

**DOI:** 10.18632/aging.202889

**Published:** 2021-04-23

**Authors:** Guohua Yu, Weiming Sun, Wansong Wang, Changhao Le, Dehuan Liang, Lang Shuai

**Affiliations:** 1 Department of Rehabilitation, The First Affiliated Hospital of Nanchang University, Nanchang 330006, China

**Keywords:** microRNA-202-3p, BMSCs, CIRI, angiogenesis, inflammation

## Abstract

Background: Cerebral ischemia-reperfusion injury (CIRI) can cause brain tissue inflammation, neuronal degeneration, and apoptosis. There is increasing evidence that microRNAs (miRNA) exert neuroprotective effects by regulating the inflammatory process during cerebral ischemia-reperfusion injury. Additionally, it is increasingly acknowledged that neuroinflammation is regulated by Toll-like receptor 4 (TLR4). However, it is unclear whether miRNA can exert its neuroprotective effects by regulating TLR4-mediated inflammation.

Methods: The effects of BMSCs over-expressing miR-202-3p on CIRI, angiogenesis in midbrain tissue, and the release of inflammatory factors (IFs) in the serum were measured using *in vivo* rat models. We also used SH-SY5Y cells to establish an ischemia-reperfusion *in vitro* cell model. The interaction between miR-202-3p and TLR4 was analyzed by overexpressing miR-202-3p and knocking down TLR4. Knockdown of TLR4 was performed using siRNA.

Results: Overexpression of miR-202-3p in BMSCs could significantly improve brain function and reduce brain damage. Simultaneously, miR-202-3p could significantly promote angiogenesis, increase the expression of vWF and VEGF, and reduce the expression of IFs. When the expression of TLR4 was significantly reduced in SH-SY5Y cells, the expression of IFs increased. Therefore, miRNA-202-3p may interact with TLR4 to modulate inflammation.

Conclusion: Our data indicated that miR-202-3p potentially exerts its neuroprotective effects and protects against CIRI by regulating TLR4-mediated inflammation.

## INTRODUCTION

Ischemic stroke is the second most prevalent cause of human death. It is associated with high morbidity, high mortality, high disability rate, and large treatment costs [1]. Cerebral ischemia-reperfusion injury (CIRI) is a key pathological contributor to the aggravation of brain dysfunction, resulting in the poor treatment and prognosis of ischemic stroke [2]. CIRI is a pathological phenomenon that refers to restoration of the blood flow supply after it has been blocked for a period during brain surgery, and is associated with increased brain dysfunction and structural damage [3]. CIRI can destroy the brain tissue structure, disrupt brain tissue function, and aggravate brain tissue damage. Moreover, CIRI can also trigger brain tissue inflammation, neuronal degeneration, and apoptosis [4].

MicroRNA (miRNA) is a very important gene-level regulatory factor, and current research on CIRI recently has been concerned with the genetic level. Many studies have reported that miRNA can play an important role in the occurrence and development of CIRI [5–7]. miRNA can cause the irreversible necrosis or apoptosis of brain cells by mediating cellular energy metabolism disorders, generating large amounts of reactive oxygen species (ROS) and causing sterile inflammation, which in turn affects brain function [8, 9].

The rat middle cerebral artery occlusion (MCAO) model to study the relationship between CIRI and miRNA is well established. Jeyaselan et al., used gene chip technology to detect miRNA expression in rat brain tissue and blood after 24 h and 48 h of reperfusion. After 24 h of reperfusion in rats, miR-103 and miR-107 expression in plasma was significantly reduced, while the expression of miR-290, -19b, and -292-5p were elevated [10]. Quantification of changes in miRNA expression in the rat cerebral cortex showed that miR-27a, -153, -129 and other miRNAs were up-regulated, while miR-20a, -320, -190, -195, -191, -23b, were down-regulated [11]. The abnormal expression of miRNA is therefore closely related to CIRI, which provides a new therapeutic direction for the clinical prevention and treatment of CIRI.

Inflammation plays a key role in ischemia-reperfusion (I/R) injury, and the nuclear transcription factor κB (NF-κB) signaling pathway is an important pathway that regulates the inflammatory response [12, 13]. CIRI can activate the NF-κB pathway and promote large-scale expression of various inflammatory factors (IFs), such as interleukin-6 (IL-6) and tumor necrosis factor-α (TNF-α). Studies also found that there are some important transcription factor binding sites in the IL-10 promoter region, such as those for NF-κB, c-Jun [14, 15].

miRNA has an important influence on the inflammatory response during I/R injury. For example, miR-21-5p can protect brain cells through the regulation of Akt signaling, and thus, inhibit brain cell apoptosis. miR-21-5p also inhibited inflammation by regulating the expression of NF-κB and inflammatory cytokines [16]. Carloni also found that miR146a, miR34a, and miR-126 participated in the inflammatory response after brain injury [17]. The aforementioned studies provided theoretical support for new treatment ideas to control inflammation during brain injury and reduce the associated blood vessel and nerve damage.

Toll-like receptors (TLRs) are important innate immune system receptors involved in the recognition of pathogenic microorganisms. They are predominantly expressed in immune cells, such as microglia, macrophages, and monocytes [18]. TLRs also exist in many other types of cells, including vascular endothelial cells (VECs), astrocytes, and neurons. TLRs on brain cells play a key role in the pathological processes underlying ischemic stroke. Recent studies have reported that TLR4 plays a deleterious role in I/R [19, 20]. Once TLRs are activated, signaling can be transduced through both MyD88-dependent and MyD88-independent pathways, resulting in activation of NF-κB, and subsequent secretion of pro-inflammatory and immunomodulatory cytokines [21, 22]. Cytokine release aids in the activation, recruitment, and adherence of neutrophils to the brain injury site, where they further contribute to the inflammatory process and apoptosis.

A recent study found that overexpression of miRNA-202-3p could activate the TGF-β1/Smad signaling pathway by targeting TRPM6, which could protect against myocardial I/R injury [23]. Studies have shown that miR-202-3p has anti-inflammatory effects [23], however, the regulatory mechanism in CIRI is poorly understood. Therefore, we hypothesized that downregulation of TLR4-mediated inflammation may be the basis of the potential protective effects of miR-202-3p. In this study, we studied the effects of miR-202-3p on MCAO animals and further confirmed that miR-202-3p was associated with the activity of TLR4.

## MATERIALS AND METHODS

### Animal experiments

Male Sprague-Dawley (SD) rats (Vital River) were housed under the following conditions (temperature 22° C ± 2° C, humidity 60% ± 10%, light time: 12 h light/dark cycles).

An MCAO model was constructed using previously reported methods [24]. Animals were divided into four groups: sham operation group, MCAO group, MCAO + BMSCs treatment group, and the MCAO + BMSCs miR-202-3p treatment group. Ten animals were conducted in each group. In the sham group, rats were only injected with normal saline (NS) through the femoral vein. In the MCAO group, MCAO rats were injected with NS. In the MCAO + BMSCs treatment group, MCAO rats were injected intravenously with BMSCs. In the MCAO + BMSCs miRNA-202-3p treatment group, MCAO rats were injected with BMSCs overexpressing miRNA-202-3p. All experimental procedures were conducted following the guidelines of the Institutional Animal Care and Use Committee of Nanchang University (Permission number: NCU-2018-1020).

### Extraction and identification of BMSCs

BMSCs were isolated from the bone marrow of rat femurs and tibias as conducted in previous studies [25], prior to observation by optical microscopy and fluorescence microscopy (DM-cil labeled BMSCs), or identification by flow cytometry according to manufacturer instruction. Cells were collected and washed twice by PBS. 5×10^6^ cells were suspended in 500 μL binding buffer including 5 μL CD45, CD34, CD106, CD29, CD11b and CD90 (Life). Then incubate cells in the dark for 20 min, and measured using flow cytometry analysis (BD FACSCanto II).

### Lentivirus transduction

To produce lentivirus, HEK 293T cells were transfected with 6.0 μg lentiCRISPR-v2 control plasmids or lentiCXCR4-gRNA-Cas9, 4.5 μg psPAX2, and 3.0 μg VSV-G plasmids using polyethylenimine reagent (PEI, Polysciences). After incubation for 72 h, the supernatants of transfected cells containing lentivirus were harvested and filtered with a 0.45μm filter. The viral titers were determined by a virus counter (Virocyt 2100). The BMSCs (1×10^5^) were transduced with the lentivirus at an m.o.i of 40. These transduced cells were incubated for 2 days and then collected for future experiments and evaluated by flow cytometry.

### Neurological function assessment

A modified Garcia test (mGarcia) on experimental rats was conducted before CIRI (day 0) and 48 h after treatment. The neurological grade was calculated as 0-18 (normal score: 18; maximum defect score: 0). Abnormal scores <18 before CIRI were excluded from the experiment.

### Haemotoxylin and eosin (HE) staining

HE staining was performed as described previously [26]. In short, brain tissue was isolated and then fixed with 10% formalin for 48 h. The optimal cutting temperature compound (OCT; Sigma) was used for tissue embedding and a cryostat was used to make 8-micron thick sections. The pictures were captured using Zeiss AxioVision (Jena).

### Brain water content

Rats were deeply anesthetized by intramuscular injection of ketamine (60 mg/kg) and xylazine (6 mg/kg) and decapitated. The drying method was used to measure the water content of the brain. After removing the cerebellar tissue, the wet weight of the left and right hemispheres was measured. The wet weight was measured using an MA110 electronic analytical balance. The brain tissue was then placed in an oven at 110° C and dried for 24 h. The dry weight of the left and right hemispheres was then measured. The Elliot formula to calculate the brain water content was used: brain water content (%) = (wet weight-dry weight) / wet weight × 100%.

### Evans blue (EB) staining

EB staining was used to assess blood-brain barrier leakage 24 h after reperfusion. First, 0.1 mL of 2% EB was injected through the tail vein, then circulated for 60 min. The SD rats were then anesthetized and euthanized after sufficient cerebral perfusion with saline. The brain tissue was taken and homogenized in PBS. After centrifugation at 15000g for 30 min, the supernatant was collected and incubated with an equal volume of 50% trichloroacetic acid at 4° C overnight. The supernatant was collected by centrifugation and measured with a microplate reader at 615 nm. Quantitative calculation was performed using a standard curve. EB content = EB amount / brain tissue weight (μg/g).

### Determination of the number of microvessels in brain tissue by immunofluorescence

Immunohistochemistry was used to calculate the microvessel density and visual field/microvessel area ratio. Immunohistochemistry was performed according to the instructions of the SP-0022 kit purchased (Bioss). Rats were anesthetized using 10% chloral hydrate, and the brain was obtained by perfusion. After fixation and dehydration, the brain tissue was cut into 4-5 μm sections for further staining and observation. Von Willebrand factor (vWF) and vascular endothelial growth factor (VEGF) are stained as brown particles in the cytoplasm of VECs. Any endothelial cells or clusters of endothelial cells stained with vWF and VEGF antibodies of a brownish-yellow color are regarded as a blood vessel. A medical image analysis system (MIAS) was used to select and analyze the peripheral and central regions of ischemia.

### ELISA for inflammatory cytokines

Briefly, 100 μL of animal serum and supernatant of cultured SH-SY5Y cells were collected and the concentrations of TNF-α, IL-18, IL-1β, and IL-6 were tested using ELISA kits (Dakewe Biotech).

### SH-SY5Y cell culture and transfection

SH-SY5Y cells are commonly used as *in vitro* models to study neuronal function. SH-SY5Y cells were cultured in 10% fetal bovine serum (FBS; Gibco), 5 mg/mL penicillin/streptomycin, 10 mL L-glutamine and 1% non-essential amino acid solution. SH-SY5Y cells were cultured in 2 mL serum-free medium for 24 h. In the absence of glucose, cells were placed in an anoxic chamber (5% CO_2_, 37° C) for 8 h, and then incubated with oxygenated medium for 25 min and 24 h under normal conditions.

siTLR4 was synthesized by GenePharma and plasmids carrying TLR4 or vector plasmid were constructed by GeneCopoeia (Rockville). The transfection was performed with lipofectamine 2000 reagent according to the manufacturer's instructions (Invitrogen). For each transfection in 24-well plates, 20 pmol synthesized RNA and/or 0.8 μg vector were used. SH-SY5Y cells were divided into five groups: control group (no I/R), I/R group, I/R + BMSCs group (BMSCs treatment), I/R + BMSCs-miR-202-3p group (BMSC-miR-202-3p treatment), I/R + BMSCs-miR-202-3p-siTLR4 group (BMSCs-miR-202-3p-siTLR4 treatment).

### SH-SY5Y cell proliferation and migration

A CCK-8 kit (Dojindo) was used to quantify cell proliferation and the OD 490nm value was obtained to determine the cell number. For migration assays, 1×10^5^ SH-SY5Y cells in 200 mL serum-free medium were placed in the top chamber of each insert (BD Biosciences). After incubation at 37° C for 36 h, cells adhered to the lower membrane were stained with 0.1% crystal violet in 20% methanol, then imaged using an inverted microscope (Olympus).

### Western blotting (WB) and quantitative real-time PCR (qRT-PCR)

Brain tissues were taken for protein lysis and Western blotting as described in the literature. We used 8% or 10% SDS-PAGE to separate protein sample (20 μg), then separated protein was transferred to PVDF membrane. After blocking with 5% BSA for 1h, primary antibodies against GAPDH and TLR4 (1:1000, Abcam) were respectively incubated with FVDF membranes overnight at 4° C. After washing with TBST for 3 times, secondary antibodies were added to the membranes and incubated at room temperature for 1 h. Protein bands on the membranes were detected by enhanced chemiluminescence and analyzed with Image-Pro Plus software 6.0. According to the manufacturer's instructions, RNA was isolated using TRIzol reagent (Invitrogen) and an RNeasy Plus Micro Kit (QIAGEN). qRT-PCR was performed using a SYBR Premix Ex TaqTM II kit (Takara). The conditions were as follows: 95° C for 10 min, followed by 30 cycles of 94° C for 30 s, 55° C for 30 s, and 72° C for 45 s. The primers used for TLR4 are listed as follows: TLR4-forward 5'-GAGGACAATGCTCTGGGGAG-3 ', reverse 5'-ATGGGTTTTAGGCGCAGAGT-3'. The primers construction were obtained from TransGen Biotech (Beijing, China).

### Statistical analysis

All experiments were conducted at least three times, and the cell experiments were repeated at least five times in each group. The results are expressed as mean ± standard deviation. Statistical analysis was performed using SPSS 22.0 statistical software package. Student's t-test and one-way analysis of variance determined statistical significance. After ANOVA for comparison between groups, we used the Bonferroni method to compare the differences between the two groups. *p*<0.05 was considered statistically significant.

## RESULTS

### BMSCs were successfully isolated and identified

**Figure 1:**
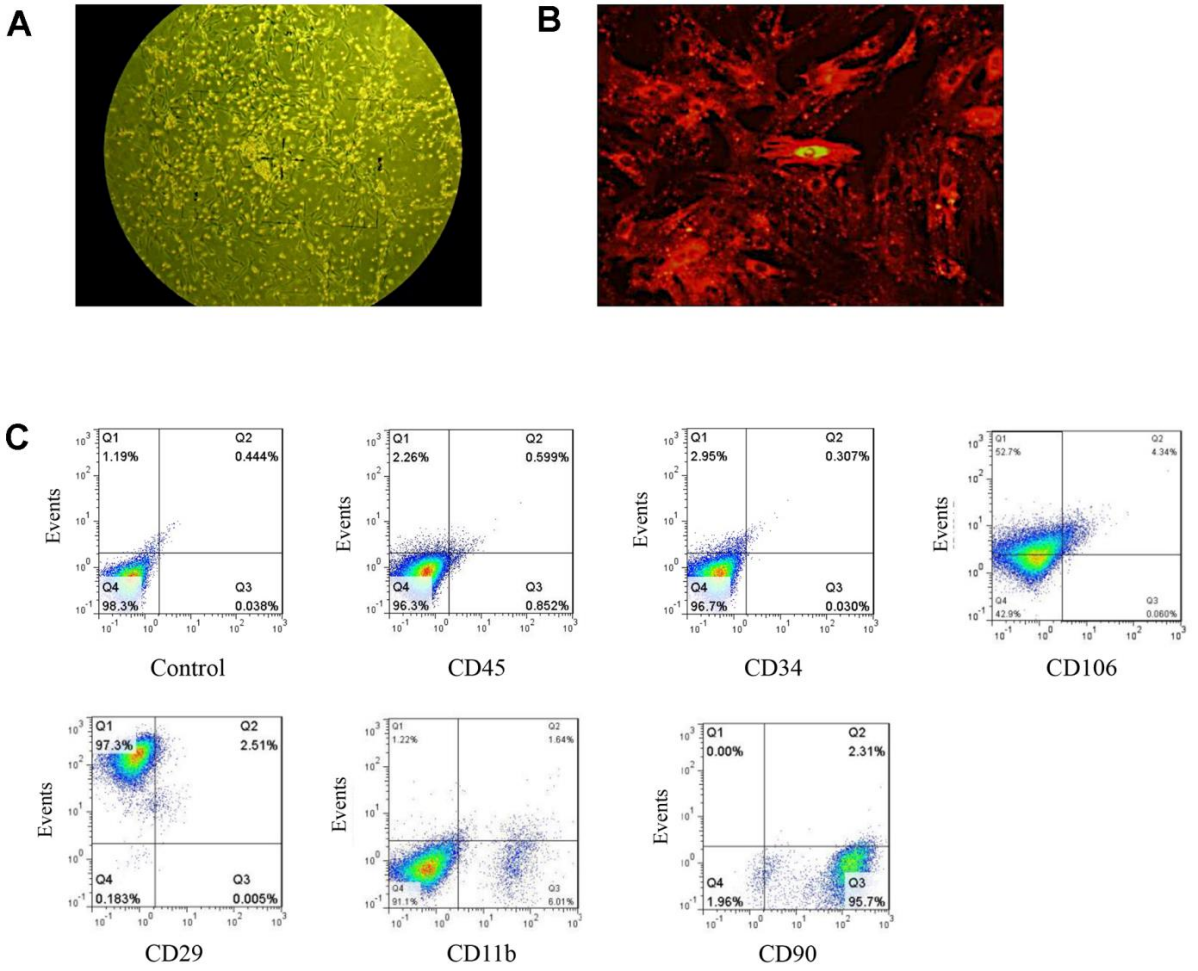
**Isolation and identification of BMSCs.** (**A**) BMSCs as observed under ordinary light microscopy (magnification: 100×). (**B**) DM-cil labeled BMSCs as observed by fluorescence microscopy (magnification: 400×). (**C**) The quantification of BMSC specific markers.

Rats BMSCs were isolated from the bone marrow of femurs and tibias. Observing BMSCs under ordinary light microscope identified that after 48 h of culture, a small number of BMSCs had adhered and cells were spindle-shaped. After 10 d of culture, BMSCs proliferated into fibroblast-like cells and had grown in clusters. When continuously cultured to the third generation, BMSCs were more uniform, displayed long fusiform morphology, and a central nucleus. (Figure 1A).

CM-DiI is a dye for short- and medium-term labeling and tracing of BMSCs. BMSCs labeled with CM-DiI showed a fibroblast-like phenotype after the 3rd passage. The morphologies were uniform and well-ordered. BMSCs labeled with CM-Dil for 24 h *in vitro* emitted red fluorescence with a 100% labeling rate (Figure 1B). BMSCs were phenotypically characterized by flow cytometry and the expression rates of CD34, CD90, and CD29 were 0.34%, 98.01% and 99.81%, respectively. The expression of CD45, CD106, and CD11b were negative (Figure 1C). The above results indicated that BMSCs were successfully isolated and identified.

### miR-202-3p overexpression in BMSCs can improve CIRI

**Figure 2:**
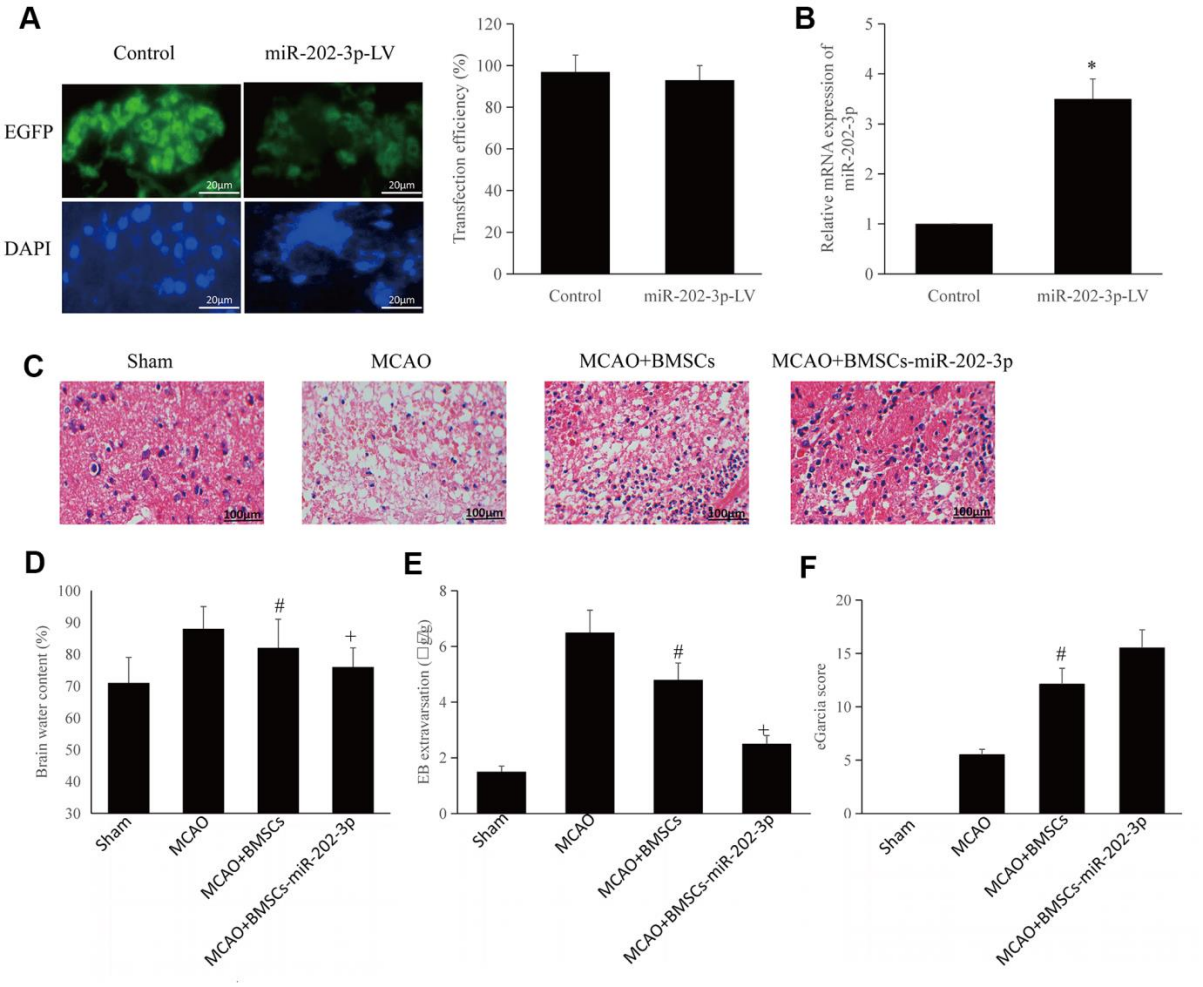
**BMSCs overexpressing miR-202-3p can improve CIRI.** (**A**) miR-202-3p was transfected into BMSCs to produce BMSCs that overexpressed miR-202-3p (magnification: 200×). (**B**) Quantification of miR-202-3p overexpression. (**C**) MCAO animal model (HE staining) (magnification: 200×). (**D**–**F**) Corresponding evaluation index. (*compared with control, ^#^compared with MCAO, ^+^compared with MCAO + BMSCs, p<0.05).

The lentiviral vectors loaded with miR-202-3p were transfected into BMSCs. We could confirm that miR-202-3p was successfully overexpressed in BMSCs (Figure 2A, 2B). The MCAO model of rats was successfully established and animals were divided into four groups: sham operation group (Sham), model group (MCAO), MCAO + BMSCs treatment group and MCAO + BMSCs-miR-202-3p treatment group (BMSCs overexpressing miR-202-3p).

The brain tissue was observed by HE staining. Brain tissue sections from the rats in the sham group were evenly stained and the blood supply could be clearly observed. The neurons in the hippocampus of the sham group were neatly arranged with complete structure, distinct layers, and nuclear non-shrinkage. Conversely, neurons in the hippocampus of the MCAO group were disordered, with significantly reduced volume, reduced cytoplasm, and nuclear shrinkage. Neurons of the MCAO + BMSCs group and MCAO + BMSCs-miR-202-3p group were arranged regularly with relatively light damage and only some cells had slight nucleus shrinkage. Compared with the MCAO group, brain tissue damage in the MCAO + BMSCs-miR-202-3p group was significantly reduced (Figure 2C).

Compared to the sham group, brain edema and EB exudation in the MCAO group were significantly increased. The MCAO + BMSCs group and MCAO + BMSCs-miR-202-3p group had reduced brain edema and permeability of the blood-brain barrier. Compared with the MCAO + BMSCs group, cerebral edema and blood-brain barrier permeability of the MCAO + BMSCs-miR-202-3p group were obviously improved (Figure 2D, 2E).

mGarcia neurological scoring system was used to detect defects in the nervous system and has been widely used to investigate early pathophysiological changes. In this scoring system, the more severe the nerve damage, the lower the mGarcia score. The score of the MCAO group was significantly lower than that of sham group and the score of the MCAO + BMSCs-miR-202-3p group were significantly higher than that of the MCAO group (Figure 2F). The above results confirmed that BMSCs overexpressing miR-202-3p had significantly improved brain function and reduced brain damage.

### miR-202-3p overexpressed in BMSCs can significantly promote angiogenesis and increase expression of vWF and VEGF

**Figure 3:**
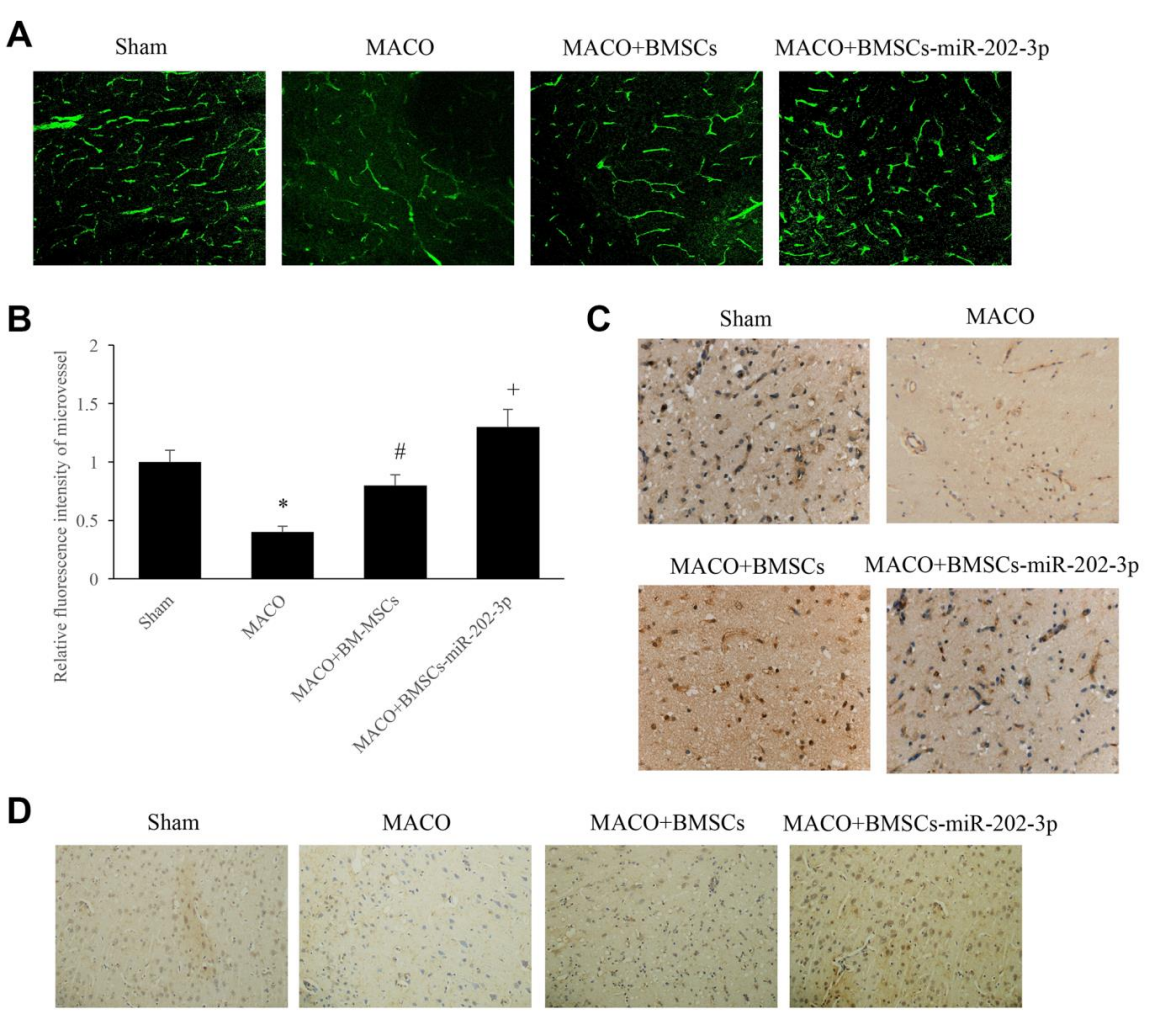
**miR-202-3p can significantly promote angiogenesis.** (**A**) Immunofluorescence detection of microvessels by injection of FITC-dextran (magnification: 200×). (**B**) Quantitative analysis of microvessels. (**C**) Immunohistochemical detection of vWF (magnification: 200×). (**D**) Immunohistochemical detection of VEGF (magnification: 200×). (*compared with sham, ^#^compared with MCAO, ^+^compared with MCAO + BMSCs, p<0.05).

Immunofluorescence detection of microvessels was performed by injecting FITC-dextran. Compared with the MCAO group, overexpression of miR-202-3p in the MCAO + BMSCs-miR-202-3p group could significantly promote angiogenesis (Figure 3A, 3B). vWF is a glycoprotein present in plasma and on the surface of VECs which is synthesized and secreted by VECs and megakaryocytes. VEGF is a factor with high specificity that promotes the growth, migration, proliferation, and increase of vascular permeability of VECs. miR-202-3p was shown to increase expression of vWF and VEGF factors in BMSCs (Figure 3C, 3D).

### miR-202-3p inhibited the release of IFs and promoted the proliferation and invasion of SH-SY5Y cells

**Figure 4:**
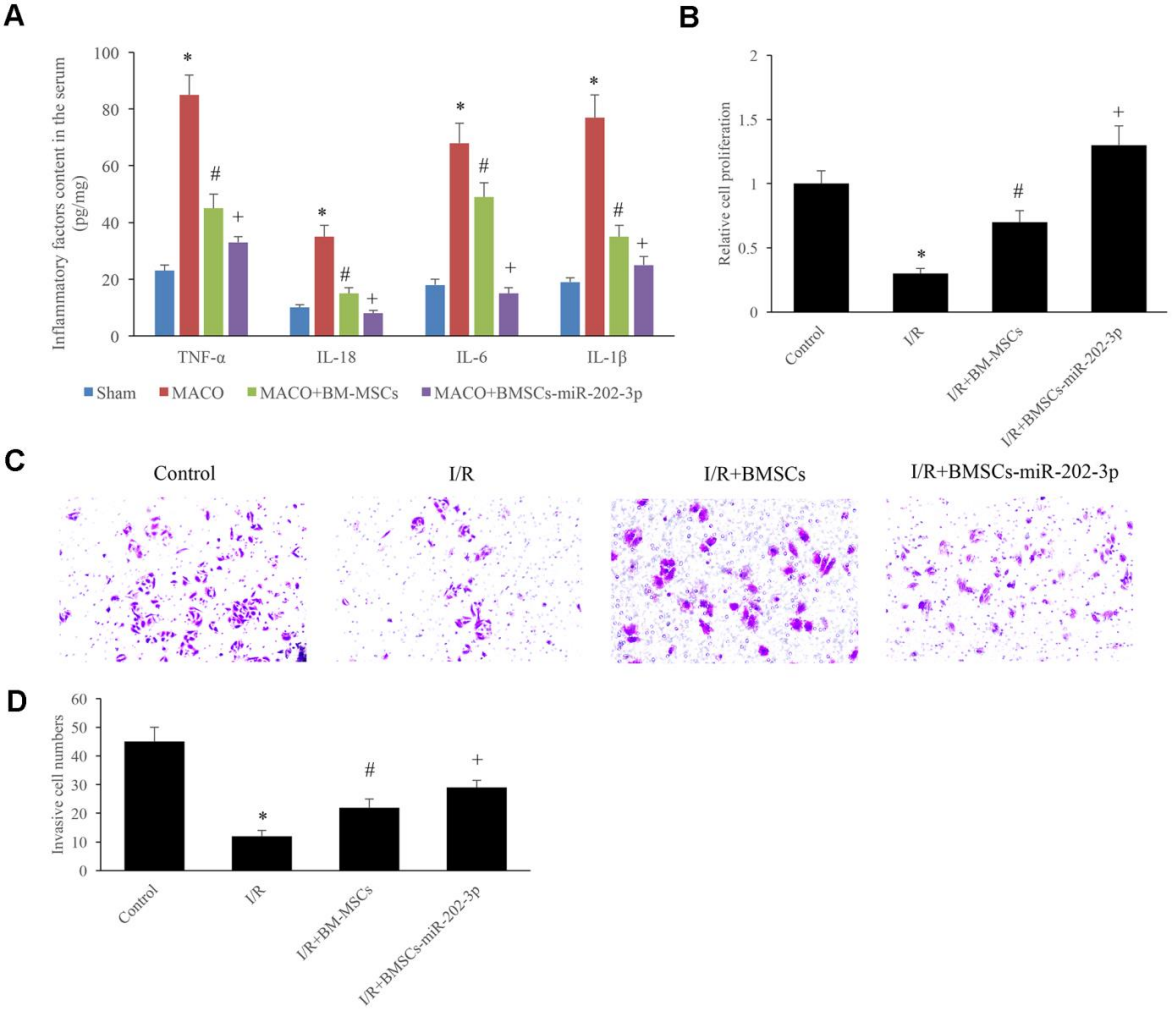
**miR-202-3p inhibited the release of IFs and promoted the proliferation and invasion of SH-SY5Y cells.** (**A**) The content of IFs in the serum. (**B**) Detection of SH-SY5Y cell proliferation. (**C**, **D**) Detection of cell invasion (magnification: 200×). (*compared with sham or control, ^#^Compared with MCAO, ^+^compared with MCAO + BMSCs, p<0.05).

Inflammatory factors (TNFα, IL-8, IL-6, and IL-1β) were detected in the serum of animals from each group. We confirmed that miR-202-3p could significantly reduce the expression of IFs (Figure 4A).

SH-SY5Y cells were used to successfully establish an I/R *in vitro* cell model. The cells were divided into four groups: control group, I/R group, I/R + BMSCs group, and I/R + BMSCs-miR-202-3p group. The experimental results showed that miR-202-3p could significantly promote the proliferation of SH-SY5Y cells (Figure 4B) and increase invasiveness of the cells invasive (Figure 4C, 4D).

### miR-202-3p significantly inhibited the TLR4-mediated inflammatory response after MCAO-induced brain injury

**Figure 5:**
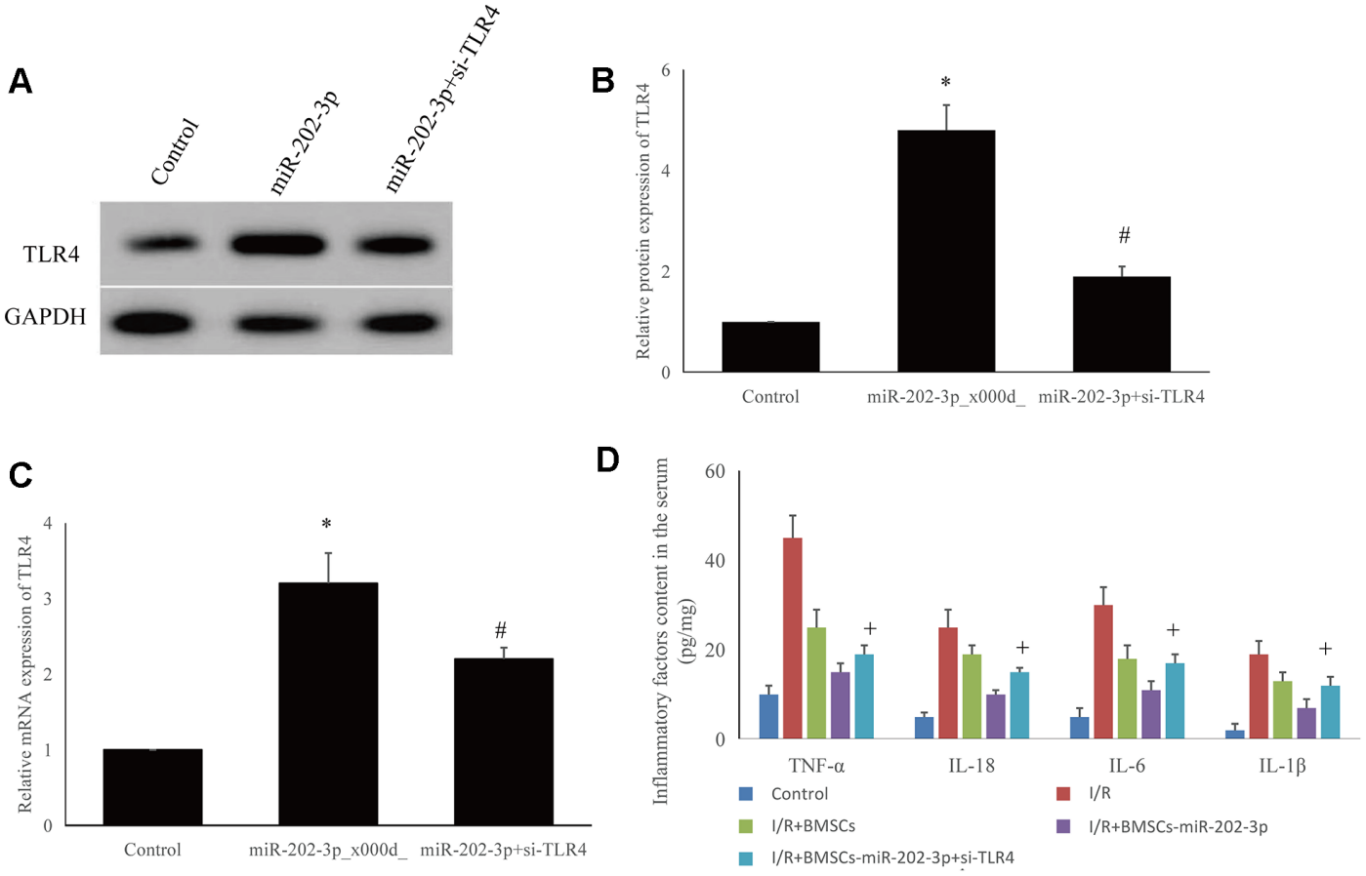
**Detection of possible targeting molecules *in vitro*.** (**A**–**C**) Overexpression of miR-202-3p could increase TLR4 expression, while si-TLR4 could reduce TLR4 expression. (**D**) si-TLR4 could reverse the effect of overexpression of miR-202-3p on the content of inflammatory factors in the supernatant of SH-SY5Y cells. (*compared with control, ^#^compared with miR-202-3p, ^+^compared with I/R + BMSCs-miR-202-3p, p<0.05).

The expression of TLR4 was significantly higher in the I/R + BMSCs-miR-202-3p group. After knocking out TLR4 in SH-SY5Y cells, mRNA and protein expression of TLR4 was significantly reduced (Figure 5A–5C).

SH-SY5Y cells were divided into five groups: control, I/R, I/R + BMSCs, I/R + BMSCs-miR-202-3p, and I/R + BMSCs-miR-202-3p-siTLR4. The expression of IFs (TNF-α, IL-18, IL-6, and IL-1β) in the I/R group were significantly increased. Compared with the I/R + BMSCs group, the expression of IFs in the I/R+BMSCs-miR-202-3p group were significantly decreased. After knocking down TLR4 in SH-SY5Y cells by siRNA, the expression of IFs was increased. Thus, miR-202-3p may target TLR4 (Figure 5D). Therefore, TLR4 may be a potential target for CIRI therapy.

## DISCUSSION

Although ischemic stroke is associated with high mortality and disability, and is one of the most common traumatic neurological diseases, current treatments are not effective [27]. Therefore, it is necessary to carry out innovative exploration of novel treatment methods. miRNA is a type of endogenous, non-coding, single-chain small RNA with a length of about 22 nt. miRNA primarily participates in the post-transcriptional regulation of genes to regulate target gene expression. miRNA plays an extremely important regulatory role in tumor development, biological development, organ formation, and metabolism [28, 29]. Previous studies on miR-202 have shown that miR-202 plays an important role in the development of cancer and the brain. Besides, despite increasing evidence that miR-202 is associated with myocardial and liver-related I/R injury, its role in CIRI is currently poorly understood [30, 31].

Studies have shown that up-regulation of miR-202-3p or knockdown of transient receptor potential melastatin 6 (TRPM-6) could reduce oxidative stress and inflammation, change cardiac hemodynamics, suppress myocardial infarction, and decrease apoptosis and myocardial fiber inhibition [23]. In this study, we found that overexpression of miR-202-3p could significantly promote angiogenesis, increase expression of vWF and VEGF factors, thereby improving brain function and reducing brain damage. In conclusion, miR-202-3p was correlated with CIRI prognosis.

TLRs are expressed in brain tissue and affect neuronal function, which also plays an important role in the occurrence and development of cerebral ischemia and secondary brain injury [32, 33]. TLR4, one of the subtypes of the TLR family, has a key role in many diseases. As TLR4 can aggravate inflammatory damage, many studies are based on directly inhibiting TLR4 expression or related signaling pathways to find suitable drugs to protect against the effects of ischemic stroke [34–36]. There is increasing evidence that down-regulation of TLR4 can significantly inhibit ischemia-induced neuronal apoptosis [37, 38]. Besides, TLR4 activation causes the release of IFs such as TNF-a, IL-6, and IL-1β. In this study, miR-202-3p could significantly reduce the expression of IFs. Subsequently, we observed that the expression levels of related IFs gradually increased with reduced expression of TLR4 in SH-SY5Y cells. These data indicated that TLR4 has a relationship with miR-202-3p.

We found that animals treated with miR-202-3p showed a significant improvement in neurological function. These data indicate that the protective effect of miR-202-3p was related to TLR4-mediated inhibition of inflammation. Although this research provided important findings, TLR4 may be just one of the many targets of miR-202-3p and there may be other molecules participate in this process.

In summary, we determined the correlation between miRNA levels and IF-related gene expression and production. We also described an interaction between miR-202-3p and TLR4-mediated inflammation, which partially explained CIRI-mediated neuroinflammation. Our results showed that the protective effect of miR-202-3p upregulation on CIRI can be attributed to the inhibition of the TLR4 pathway and resultant reduction in the inflammatory response. Our data suggested that miR-202-3p is a potential therapeutic target for CIRI. However, whether miR-202-3p treatment is effective in improving the clinical outcome in patients requires further study.
